# Development of Robust Behaviour Recognition for an at-Home Biomonitoring Robot with Assistance of Subject Localization and Enhanced Visual Tracking

**DOI:** 10.1155/2014/280207

**Published:** 2014-12-21

**Authors:** Nevrez Imamoglu, Enrique Dorronzoro, Zhixuan Wei, Huangjun Shi, Masashi Sekine, José González, Dongyun Gu, Weidong Chen, Wenwei Yu

**Affiliations:** ^1^Graduate School of Engineering, Chiba University, Chiba 263-8522, Japan; ^2^Research Center for Frontier Medical Engineering, Chiba University, Chiba 263-8522, Japan; ^3^Department of Electronic Technology, University of Seville, 41012 Seville, Spain; ^4^Institute of Robotics and Intelligent Information Processing, Department of Automation, Shanghai Jiao Tong University, Shanghai 200240, China; ^5^Department of Biomedical Engineering, Shanghai Jiao Tong University, Shanghai 200030, China; ^6^Spanish National Research Council, Bioengineering Group, 28500 Madrid, Spain

## Abstract

Our research is focused on the development of an at-home health care biomonitoring mobile robot for the people in demand. Main task of the robot is to detect and track a designated subject while recognizing his/her activity for analysis and to provide warning in an emergency. In order to push forward the system towards its real application, in this study, we tested the robustness of the robot system with several major environment changes, control parameter changes, and subject variation. First, an improved color tracker was analyzed to find out the limitations and constraints of the robot visual tracking considering the suitable illumination values and tracking distance intervals. Then, regarding subject safety and continuous robot based subject tracking, various control parameters were tested on different layouts in a room. Finally, the main objective of the system is to find out walking activities for different patterns for further analysis. Therefore, we proposed a fast, simple, and person specific new activity recognition model by making full use of localization information, which is robust to partial occlusion. The proposed activity recognition algorithm was tested on different walking patterns with different subjects, and the results showed high recognition accuracy.

## 1. Introduction

At-home biomonitoring systems have become an important solution in the medical field as an assistive technology for the people who have difficulties leaving their houses, such as elderly people or motor-function impaired persons (MIPs) [1–8]. Benefits of these systems include more convenient and comfortable ways of healthcare for patients and reduction in the workload of the therapists [5–8].

At-home healthcare applications cover a wide range of topics regarding physiological measurements or biomonitoring applications. Biomonitoring research in at-home health care emerged from the demand on improving the quality of life (QoL) of the people, such as to measure the vital signs, check their health over through the measurements, identify abnormalities from long-term at-home observation, and monitor rehabilitation assistance.

In this research, our concern is mostly focused on the observation and analysis of motor function-related daily activities of the people in need such as the elderly and the motor-function impaired people. For this reason, tracking the subject and detection of daily activities are crucial tasks to be performed. The system should record data of continuous walking patterns for further analysis by the experts or computer-aided diagnosis systems. Therefore, in this study, we put our primary concern on tracking of subjects and detection of daily activities at-home with a mobile robot as an assistive technology to support the people with motor-function impairment.

There are several approaches for daily observation that provide information about the activities that the subjects are performing [9–16]: (i) wearable sensors that include a wide range of different sensors such as accelerometers and (ii) smart house systems that implement solutions with multiple vision devices attached to certain fixed sites in a house. The main advantages of wearable systems are that they provide cheap and accurate solutions for activity recognition and analysis. The complexity of these systems, regarding the recognition algorithms and maintenance, is lower than other solutions such as smart houses with multiple-camera systems. However, the main disadvantage of wearable sensors is the attention required by the users. Subjects can be bothered by the fact that they have to wear many sensors and they have to be careful to avoid damaging the sensors while doing daily living things: sitting, eating, sleeping, and so forth. Quite often, subjects have to put on and off and charge the batteries of the sensors.

On the other hand, smart houses do not have these disadvantages; daily activities can be tracked by systems that include multiple observation devices such as motion tracking systems, cmos/ccd cameras, and color and infrared cameras such as Kinect [8, 10, 11, 17, 18]. For instance, Tamura et al. [7] proposed an at-home biomonitoring system in which automated electrocardiogram (ECG) signals are measured and observed in some of the arranged places at home, such as bed, toilet, or bathroom, without using body surface electrodes. Even if they provide reliable observation capabilities to detect and analyze the daily activities performed by the subjects, they also have some disadvantages. Due to the number of observation devices, these systems are expensive and hard to set up and maintain. Moreover, despite the large number of sensory devices, it is still possible to have blind spots in indoor environments that can prevent the monitoring.

As proposed in our previous works [5, 8, 19, 20], our main idea is to use assistive robotics to support elderly or motor-function impaired persons biomonitoring. Although a mobile system brings more complexity to handle daily observation tasks, a mobile robot solution includes several advantages: (i) it is a cheaper smart system than smart houses, (ii) it could make full use of the capability of sensors, since the sensors could be brought to a position and an angle optimal or suboptimal to observation, (iii) it avoids the multiple vision modules by using a mobile monitoring system, and (iv) it prevents the need of the subjects for wearing any sensors [5, 8, 19, 20] compared to wearable sensor applications. These advantages make the mobile robot a good candidate for assistive technologies. In addition, based on the Robocare project, Cesta et al. [21] made a preliminary experiment about the way that people evaluate the use of a robot at home for different purposes such as surveillance, service, or companion tasks [21, 22]. In most cases, the robot and subjects had some interactions considering the tasks for each scenario [21] such as accepting voice commands from subjects and serving. In these experiments, with 40 elderly subjects, activities that are usual to occur during the daily living were investigated [21]. After making a questionnaire to the experiment subjects, they gave a positive response [21] and supported the idea of having mobile robotics applications for at-home assistive tasks.

### 1.1. Background

**Figure 1:**
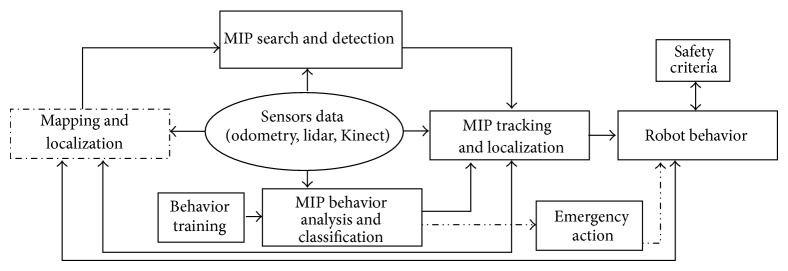
Required modules to implement an at-home biomonitoring mobile robot for the support of the elderly or MIPs.

Therefore, in our previous research [5, 8, 19, 20], we presented an at-home biomonitoring mobile robot project to improve the quality of life of motor-function impaired persons (MIPs). The robot observes the subject and recognizes the activity he is performing. It is also able to provide analysis of walking pattern if the lower limb joint trajectories were traceable [5, 8]. To achieve these tasks, there are several modules that have to be developed.  Figure 1 presents the scheme of these modules.

Until this moment of our current research, we have focused on some of these modules. Due to the low resolution image, high illumination changes and low texture details, for our real time applications, and color-based trackers supported by the depth data are the most suitable options. So, we developed a visual tracking model in [20] using an improved color tracking algorithm that takes advantage of particle filter model, depth data, and bottom-up saliency map [20]. Saliency maps are the visual attention computational models inspired by the visual system [20]. The idea is to identify attentive regions on the scene by reducing to redundancy which improved visual tracking in [20] when combined with the particle filter color tracker. Particle filter based color tracker is a robust tracker; however, there are many parameters to consider; the motion of the robot should be integrated to particle motion model to increase the visual tracker performance, which is very difficult to implement because of the indeterministic and uncertain behaviour of the robot that depends on the subject position. For the robot behaviour, in most of the cases, we have made experiments using a reactive control model [5, 8, 19] for the robot movement while tracking the subject of interest. However, without having a global reference of robot and subject position, it is very difficult to implement a robust mobile tracking system. By knowing the global position, it is possible to have different options for the movement of the robot using path planning algorithms.

In addition, activity recognition regarding the MIP behaviour analysis and classification (see Figure 1) was accomplished by analyzing the features from the human body contour. This was done by extracting height and width ratios from the several body parts from the binary images of extracted subject tracking regions, and then, Hidden Markov model (HMM) [5, 8] was used to classify the current action by observing the changes in the body structure by using the joint angles of the lower limb [5, 8]. Moreover, normal walking and impaired walking were differentiated by HMM-based classification with high accuracy. However, the problem with the features used in [5, 8] is that it is not robust when there is partial occlusion of the lower limb part of the body. Lower limb part of the body is crucial to discriminate between walking and standing during the activity recognition process. Also, this algorithm requires specific training models for each subject separately, so it is hard to obtain good activity detection results, especially for dynamic activities such as walking, for several subjects by using only one trained model.

Due to the problems stated in the previous versions of our work [5, 8, 19, 20] several improvements and experimental analysis have been done for the robot behavior and subject tracking.

- First of all, instead of using reactive control, robot operating system (ROS) [23] was integrated to our system as many works also take advantage of ROS for the mobile robot applications [23–26]. The main advantages of using ROS are the global mapping and localization [23–26] functionalities that increase the performance of the robot motion, provide more flexibility to robot during subject tracking, and enable the use of a more robust activity recognition approach under occlusion cases.
- Then, we have changed depth and saliency improved particle filter color tracker with a new color tracking approach presented in [27]. This approach mainly changes the particle filter model with scale and orientation adaptive mean shift tracker (SOAMST) [27] since it also provides good tracking results even under scale and orientation changes [27] with less parameter adjustments. Also, depth integration [19, 20] is also done for the SOAMST algorithm too to improve the robustness of visual tracking.

### 1.2. Contribution

Despite several stated improvements on our robot structure, the main contribution of this paper is summarized in the following points.

- To be able to achieve robust robot tracking and activity recognition, visual tracking should be satisfactory enough to find subject on the scene in changing environmental conditions. For example, different light conditions, which are also affected by the pose and distance between subject and robot, can affect the color information that can lead to possible false tracking. So, we have researched how illumination changes affect the visual tracking, which is crucial in daily at-home biomonitoring. By visual tracking analysis with different illumination conditions, it is possible to determine which lux (quantitative measurement of the light conditions) values provide better or worse tracking results. Then, robot tracking can be improved with the consideration of these constraints, and also, as a future work, the robot would be able to switch between depth-based and color-based tracking to increase the visual tracking performance.
- There are many works on the analysis of navigation and path planning for mobile robot and how the environmental layout affects the behaviour during navigation [28–30] without the consideration of continuous subject tracking. But there are not studies focused on analyzing the robot motion related parameters for robot based subject tracking regarding the visual tracking constraints such as illumination and distance, environmental settings, smoothness of the tracking path of the robot, and subject safety. Therefore, in this work, several experiments were done to analyze and find the suitable parameters for our indoor biomonitoring robot task.
- With the integration of localization and mapping algorithms, we proposed a new localization assisted activity recognition algorithm, which suggests a fast model and is robust to occlusion cases where the lower limb body is not visible. A state machine-based activity recognition approach is used, where global location, the height, and ratio of the upper body part within a defined region of the subject are used. There is no need of using training data because it utilizes person specific heuristics to classify the activity of the subject by considering activity state changes.

The paper continues as follows.  Section 2 describes the robot platform including both hardware and software components considering ROS and its integration to system, visual and robot based tracking, and activity recognition.  Section 3 gives the visual tracking tests and the details for illumination change based experiments on color based visual tracking. Then, Section 4 shows the experimental analysis with robot behaviour with consideration of illumination constraints obtained by visual tracking analysis with different lighting conditions during subject tracking with different parameters for robot path planning.  Section 5 demonstrates the experimental results on the proposed activity recognition algorithm. Finally, discussion and conclusion remarks are given.

## 2. System Architecture

**Figure 2:**
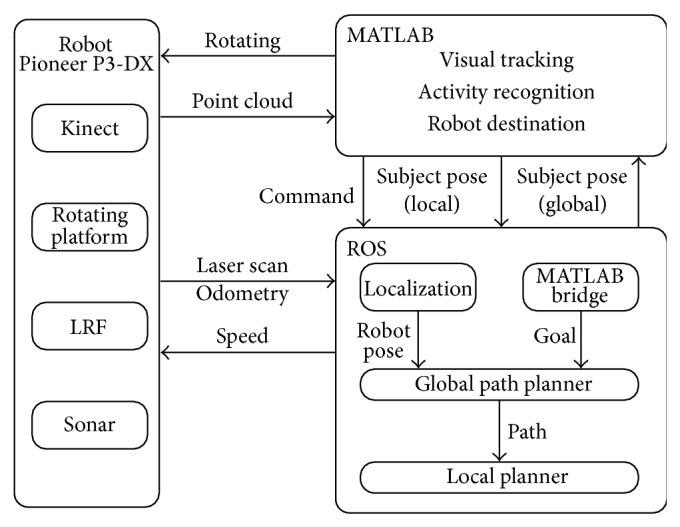
System structure including hardware and software components.

The system structure is given in Figure 2, where all the components, hardware and software, of the system are presented. The hardware is composed of the robot platform and its components. The software part includes the ROS [23, 25] and algorithms in MATLAB codes to perform a given task such as visual tracking, robot tracking, and activity recognition.

### 2.1. System Hardware

**Figure 3:**
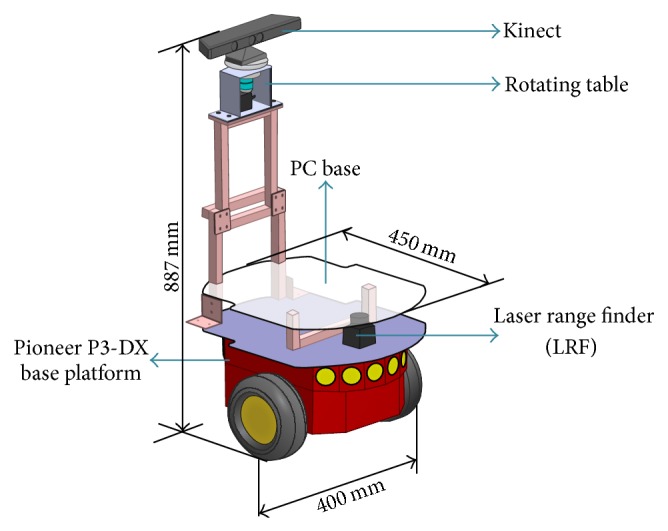
Mobile robot hardware structure.

**Figure 4:**
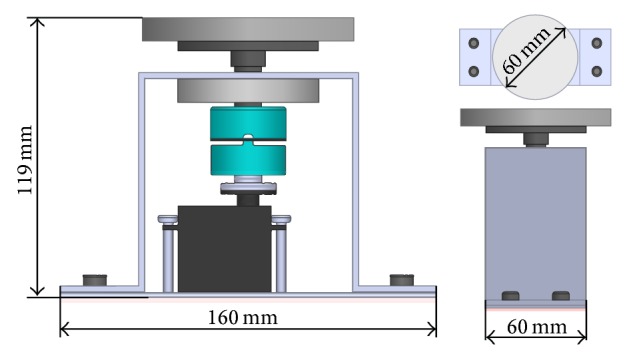
Rotating table.

The robot hardware (Figure 3) is assembled base on Pioneer P3-DX, equipped with a laser range finder (LRF) and a Kinect sensor on a rotating platform (Figure 4). The LRF is used for mapping, localization, and obstacle avoidance. The Kinect is used for subject detection and it is mounted on a rotating platform in order to extend the detection range so that the visual tracking can be achieved even if there is the difficulty for the robot to change the pose.

### 2.2. System Software

The system uses ROS for robot behavior control and MATLAB for an improved visual subject tracker, activity recognition, and robot command control.

### 2.3. ROS Integration into the System

ROS is used for mapping, localization, and robot movement (to a point established by MATLAB) and converts the local subject pose to the global subject pose. In detail, the ROS communicates with the robot using the ROSARIA package [23]. The communication purpose is to have control of the robot and receive odometry data. To control the robot, MATLAB sends three types of commands: rotating with an angle, going to a goal, and stopping (shown as robot destination task in Figure 2), and the communication between ROS system and MATLAB is achieved by using MATLAB engine.

**Figure 5:**
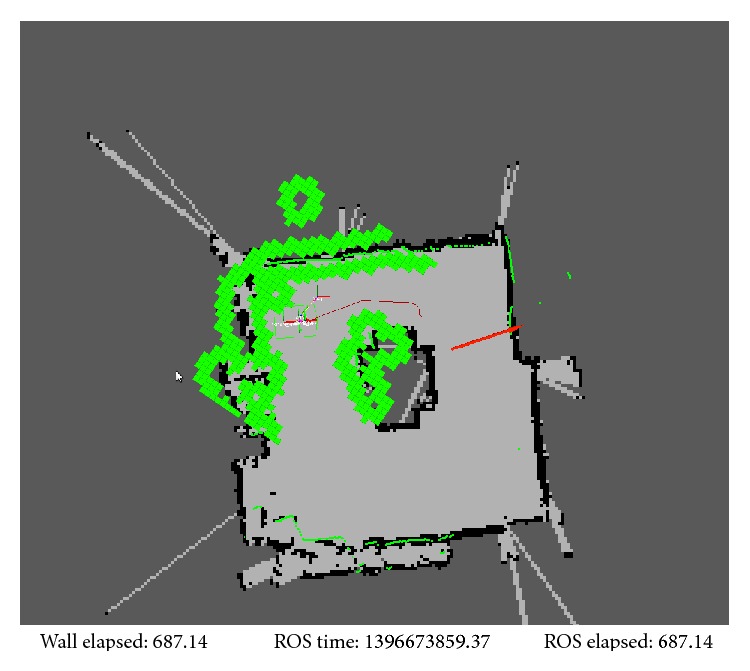
ROS localization and path planing on the global map.

As for the control of the robot movement that allows sending the robot to a specific point, a navigation system is implemented in ROS. First, we manually control the robot to explore the surrounding environment and use Gmapping package [24, 25] to map the environment by combining the information of LRF and odometry (see Figure 5). From this point, ROS navigation stack, including localization, costmap, global path planner and local planer, is used for autonomous robot motion planning. Adaptive Monte Carlo localization approach (AMCL) [26] is used to locate the current pose of robot, based on the map built by Gmapping. The costmap [31] is an occupancy grid map, which keeps all the obstacle information, including the static map and dynamic updating information provided by robot sensors. The cost of each grid is related to the distance between the robot and an obstacle. For example, when the robot is closer, the cost is bigger.

According to the current position of robot, the destination point is established by MATLAB and the costmap, and a Dijkstra's algorithm based global path planner is implemented to plan a shortest feasible path. Trajectory rollout [32] and dynamic window approach (DWA) [33] are used to generate the velocity commands that are sent to the robot, in order to follow the path generated by global path planning until the robot arrives to the destination or it is stopped by MATLAB command. Since the costmap contains the obstacle information, the global path planner and local planer can avoid the obstacles automatically (see Figure 5).

### 2.4. Visual Tracking

The visual tracking algorithm is implemented on MATLAB by using the scale and orientation adaptive mean shift tracking (SOAMST) algorithm [27]. We made improvement by integrating Kinect depth image support to increase the importance of weights based on the depth likelihood as proposed in [19, 20]. In addition, saliency map integration is also possible by updating the weights as proposed in [20], but in this work, we omitted using saliency for real-time processing performance. The details of the SOAMST color region tracker can be find in [27], and depth and saliency improvement details can be implemented similar to the [19, 20].

Depth improved SOAMST is explained as follows [19, 20, 27].

**Figure 6:**
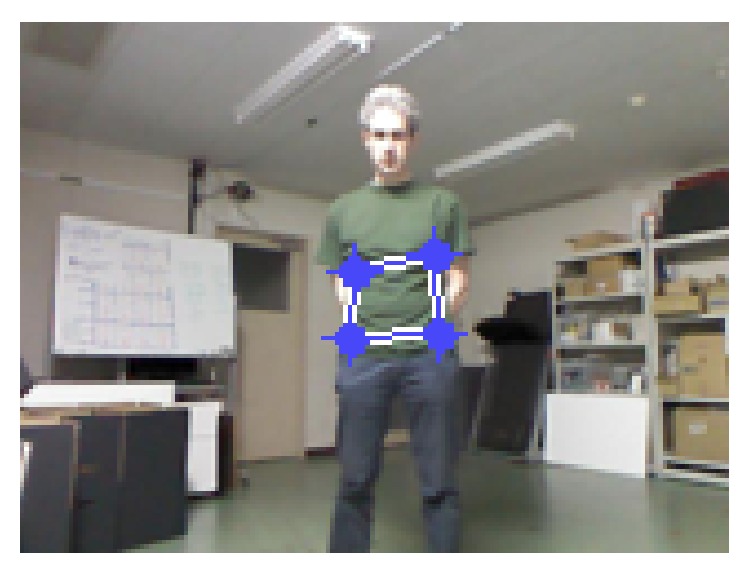
Manually selected region.

(a) Initial target model is calculated by manually selecting the region of interest (Figure 6) on the subject based on normalized color histogram from R, G, and B color channels with 16 bin quantization, and initial target position (*y*_0_) is decided by the target model.

(b) Based on the initial target position, initialize the target depth or distance to the robot from depth data *D* as follows:

(1) $$\begin{array}{c} \text{tempDepth}=D\left(y\_0-5:y\_0+5,x\_0-5:x\_0+5\right);\\ d_{0}=\text{round}\left(\text{median}\left(\text{double}\left(\text{tempDepth}\left(∶\right)\right)\right)\right). \end{array}$$

**Figure 7:**
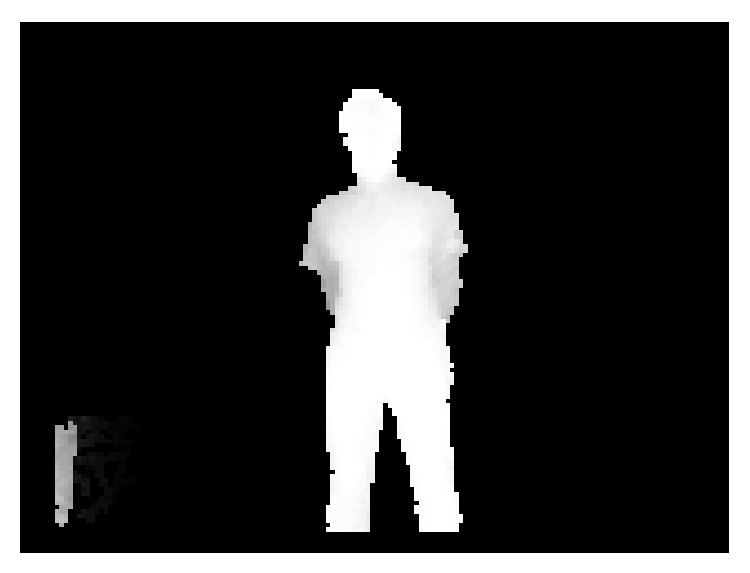
Depth likelihood map.

(c) Set iteration number, *k*, to 0.

(d) For the current depth map, based on the previous target position, *y*_0_, find the depth likelihood map (Figure 7) as in (2), in which reference target region point will have the highest likelihood to the points at current frame with similar depth:

(2) $$\begin{array}{c} P_{y}=\frac{1}{\sigma \sqrt{2\pi }}\exp\left(\frac{-{\left(D_{y}-d_{0}\right)}^{2}}{2\sigma^{2}}\right), \end{array}$$

where *P*_*y*_ is the depth likelihood map calculated by the depth map *D*_*y*_ based on the depth values at point *y* and *d*_0_ is the target depth value at previous frame.

(e) For the current data based on the previous target position, *y*_0_, find the target candidate model.

(f) Calculate the weights of each pixel in the target candidate region as

(3) $$\begin{array}{c} w_{i}=d_{i}\overset{m}{\underset{u=1}{\sum }}\sqrt{\frac{{\hat{q}}_{u}}{{\hat{p}}_{u}\left(y_{0}\right)}}, \end{array}$$

where *d*_*i*_ is the depth likelihood value obtained from *P*_*y*_ and *q*_*u*_ and *p*_*u*_(*y*_0_) are the reference target model and target candidate model of the search region.

(g) Then, new target position can be calculated as

(4) $$\begin{array}{c} y_{1}=\frac{\sum_{i}x_{i}w_{i}}{\sum_{i}w_{i}}, \end{array}$$

where *y*_1_ is the new matched point for the subject position obtained by comparing the initial target model and current frames candidate model, *x*_*i*_ is the points in the search region, and *w*_*i*_ is the weights at *x*_*i*_.

(h) This operation continues until matching point converges to a value as in

(5) Δd=y1−y0y0=y1if  Δd<ε || k≥15if  Δd<go  to  step ielseif  Δd<k=k+1;if  Δd<go  to  step e.

(i) After the new tracking point is found, the height, width, and orientation of the target candidate model can be calculated by singular value decomposition of the covariance matrix obtained by the second order moment features as follows:

(6) $$\begin{array}{c} \left[\begin{array}{cc} u_{11} & u_{12}\\ u_{21} & u_{22} \end{array}\right]\times \left[\begin{array}{cc} a^{2} & 0\\ 0 & b^{2} \end{array}\right]\times {\left[\begin{array}{cc} u_{11} & u_{12}\\ u_{21} & u_{22} \end{array}\right]}^{T}=\text{SVD}\left(\text{Cov}\right), \end{array}$$

(7) Cov=μ20μ11μ11μ02 a=kλ1b=kλ2 U=u11u12u21u22,

where ${\left[\begin{array}{cc} u_{11} & u_{21} \end{array}\right]}^{T}$ and ${\left[\begin{array}{cc} u_{12} & u_{22} \end{array}\right]}^{T}$ can yield the orientation of the tracked region and *a* ^2^ and *b* ^2^ are the scaled eigen values of Cov in (7), which can represent the height and width of the tracked ellipsoid region.

(j) Then, next step is to find next search region for the next frame as

(8) $$\begin{array}{c} {\text{Cov}}_{2}=U\times \left[\begin{array}{cc} {\left(a+\Delta d\right)}^{2} & 0\\ 0 & {\left(b+\Delta d\right)}^{2} \end{array}\right]\times U^{T}, \end{array}$$

(9) $$\begin{array}{c} \left(x-y_{1}\right)\times {\text{Cov}}_{2}^{-1}\times {\left(x-y_{1}\right)}^{T}\leq 1, \end{array}$$

where the points *x* that provide the conditions in (9) can be in the next search region. Δ*d* is the change for the search region in the next frame.

### 2.5. Activity Recognition

**Figure 8:**
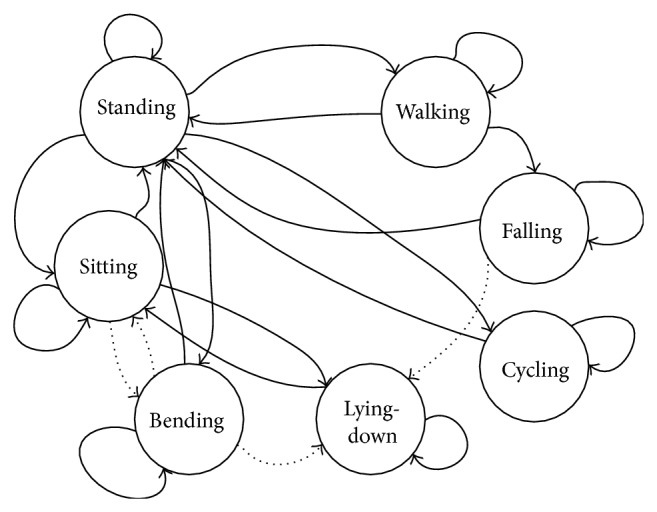
State transition applied in activity recognition model.

By the integration of mapping and localization, new activity recognition has been proposed to handle the problems of our previous approach such as occlusion cases [5, 8]. This update includes a new, fast, and person specific model that does not require any training process using high amount of off-line training data. The new model takes advantage of person specific heuristics, state transition rules, and global localization of the subject by utilizing the information through robot localization. Using these features, the model classifies the possible indoor daily activities such as walking, standing, sitting, bending, lying down, cycling, and falling. The state transition rules between activities are given in Figure 8.

Person specific features include variables such as

**Figure 9:**
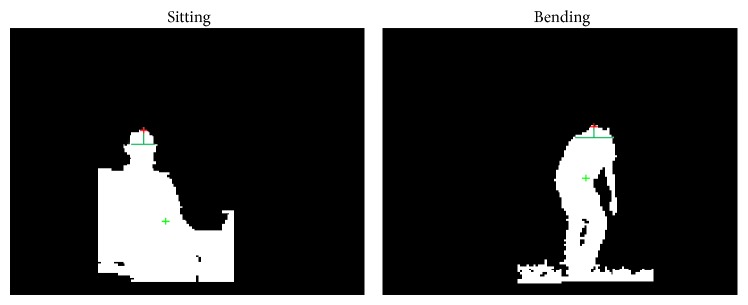
Extracted contour region, lines on the top part of the body to calculate the ratio parameter, and tracking point on the extracted region.

(1) height parameter based on the extracted contour region of the tracked subject;
(2) change in the height parameter between frames;
(3) a ratio parameter that can be calculated through top region of the extracted body contour (see Figure 9);
(4) subject location based on the tracking point on the subject calculated by the visual tracking algorithm using Kinect and robot global position obtained by lidar and odometry (The transformation from local Kinect 3D data to global 2D map is given in (10) [34]:
  (10) $$\begin{array}{c} \left[\begin{array}{c} S_{Gx}\\ S_{Gy} \end{array}\right]=A\times \left[\begin{array}{c} S_{Lx}\\ S_{Ly} \end{array}\right]+\left[\begin{array}{c} T_{Gx}\\ T_{Gy} \end{array}\right], \end{array}$$
  (11) $$\begin{array}{c} A=\left[\begin{array}{cc} s_{x}\cos\left(\alpha \right) & -s_{y}\sin\left(\alpha \right)\\ s_{x}\sin\left(\alpha \right) & s_{y}\cos\left(\alpha \right) \end{array}\right], \end{array}$$
  (12) $$\begin{array}{c} T_{Gx}=R_{Gx}-d\cos\left(\theta \right) \end{array}$$
  where *S*_*Gx*_ and *S*_*Gy*_ are the subject's global *x* and *y* positions on global 2D map and *S*_*Lx*_ and *S*_*Ly*_ are the local Kinect data of the subject on the relevant *x*-axis and *y*-axis of Kinect to global 2D map. *α* is the Kinect pose on global map calculated by the robot pose and rotating table angle. The transformation matrix, **A**, is shown in (11), and *s*_*x*_ and *s*_*y*_ of **A** are the scaling coefficients on *x*-axis and *y*-axis. *T*_*Gx*_ and *T*_*Gy*_ are the translation values on *x*-axis and *y*-axis, respectively, due to the difference between robot center and Kinect position on the robot. Translation values were computed as in (12) with the given *R*_*Gx*_ and *R*_*Gy*_, which are the robot positions in global 2D map, and *d* is the distance between Kinect position and the robot center. And, the robot pose on global map is given as *θ*.);
(5) velocity of the subject between a defined number of frames.

As it has been mentioned that the new model can be used to classify static activities such as standing, sitting, and lying down. However, features to represent static activities are not enough to detect dynamic or location based specific behaviors such as cycling on a stationary bicycle. Hence, we take advantage of the subject global position on the 2D map defined in (10). If the subject is sitting or bending at a specific global map position (marked as the stationary bicycle zone), it can be inferred that the subject is using cycling machine. And, if the extracted body contour is changing its global position on the global map and the height of the extracted region is for the standing condition, it can be inferred that the subject is walking even if the subject body is not fully extracted or there is partial occlusion.

As long as visual tracking and localization of the subject are successfully done, activity recognition model can achieve the classification task with high accuracy independent of posture and occlusion cases.

Since the one purpose of the project is the biomonitoring of the human walking conditions for the different types of situations, such as normal, impaired, and elderly walking patterns, it is important to for the system to detect the walking activity for the stated different walking patterns. Because it is important to know the walking activity for the biomonitoring system to record the data of the subject's walking behaviour, especially for the people in demand such as elderly or impaired people. By this way, experts or an algorithm can analyze the recorded data whenever necessary.

There are many algorithms that can detect walking activity, but most of the experimental results are generally based on normal walking conditions including other daily activities. Therefore, in this work, we tested the proposed activity recognition of algorithm for the walking condition on normal, impaired, and elderly walking patterns. Experiments showed that the proposed algorithm can robustly detect walking activity for the various patterns as long as the subject tracking is accurate. The results for the walking activity for different patterns can be seen in the experimental results section.

To demonstrate how the state transitions were handled, state transition rules for standing and walking activities, which are representative activities in terms of their frequency of occurrence and dependency on localization information, were described as in Algorithm 1.

In Algorithm 1, *States*_*t*_ is the activity on frame *t*. Activity labels is defined as follows: (i) standing = 0, (ii) walking = 1, (iii) sitting = 2, (iv) bending = 3, (v) cycling = 4, (vi) falling = 5, and (vii) lying  down = 7. *t* is the current frame number. *S*_Fall_ and *S*_NoFall_ are the speed parameters to decide fall or non-fall cases based on the tracked subject height at the current and previous frames as *Height*_*t*−1_^*s*^ and *Height*_*t*−1_^*s*^. *S*_Walking_ is the subject speed threshold to decide whether the subject is moving or not. Localization assistance uses the global subject position *P*_*t*_^*s*^(*G*_*x*_, *G*_*y*_) for the necessary cases. And, *r*_*t*_ is the ratio parameter at frame *t* defined around the maximum height of the tracked region as shown in Figure 9 to discriminate activities such as sitting, bending, and lying down based on person specific* Ratio* threshold.

## 3. Experiments of Visual Tracking Performances and Effect of Lighting Conditions

Obviously, it is crucial factor for the monitoring and activity recognition process to have good robot motion and robust visual tracking performance. Therefore, initially, this work is also focused on two other aspects: (i) visual tracking limitations occurs due to the color model changes on the tracked subject, in which color model is affected by the illumination conditions from the light source or the distance between robot and subject, and (ii) then the experiment and analysis of robot behaviour with the given environmental conditions including the visual tracking constraints. Finally, after the system tests improvements on robot tracking, the proposed activity recognition is tested on various walking patterns to check its robustness to subject and pattern variations.

### 3.1. Depth Improved Visual Tracking Experiments

First of all, we have tested the SOAMST [27] and depth improved SOAMST tracking model proposed in this work in Section 2.3. The experiments were done by using the recorded data described in Table 1 that includes 6000 frames with various walking patterns and color representations from different subjects.

**Figure 10:**
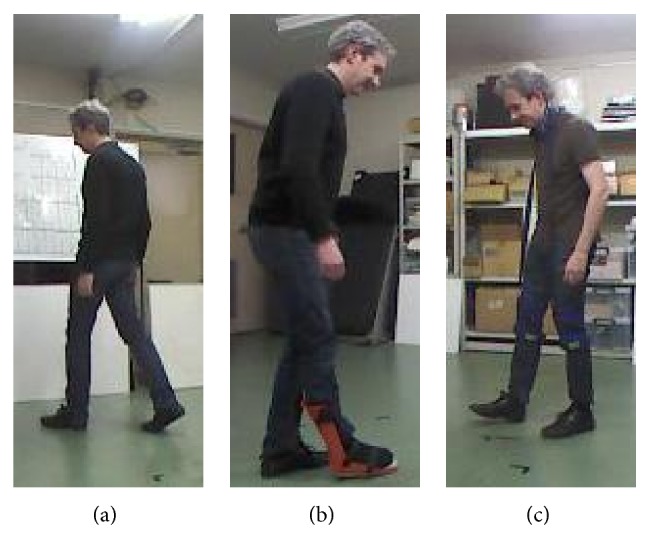
(a) Normal walking, (b) simulated impaired walking, and (c) simulated elderly walking.

In this study, the same subjects were required to test elderly, impaired, and normal walking patterns to see how the walking patterns from the same subject differ during tracking and activity recognition tasks. Therefore, each subject participated in experiments to record data on elderly, impaired, and normal walking situations. Normal walking experiments were done with self-paced walking speed, without any limitations (Figure 10(a)). On the other hand, the elderly walking and impaired walking cases were simulated with a wearable simulation kit as shown in Figures 10(b)-10(c). The impaired walking kit (Figure 10(b)) was used on the right foot of two right footed subjects to cause impairment on the right lower limb. And, for the elderly walking, the kit causes walking difficulty due to tied joint in a way that neck and knees were constrained to cause difficulty on standing straight and making big moves as in normal walking. Therefore, the two simulating kits resulted in quite realistic walking patterns for both the elderly and impaired walking cases.

Experimental data recorded in Table 1 is prepared with stable light conditions by opening all light sources in the room to be able to see the improvement by the proposed depth likelihood integration to SOAMST color region tracker while decreasing the effect of illumination changes.

Table 2 gives the number of frames with correctly tracked subjects and false trackings for each recorded data. For the SOAMST, we have seen that good tracking performances can be obtained when the color of the subject representation has high contrast with the background condition. On the other hand, color region tracker is not robust enough when the foreground and background representations are similar to each other. Also, high illumination variations or shadows on the foreground region may result in false tracking cases with the SOAMST [27] algorithm.

However, proposed depth integration improved the tracking accuracy a lot, in which tracking was accomplished without any false tracking in five of the six cases of the dataset. For the proposed depth improved SOAMST, only one case yielded worse result than SOAMST [27] using only color model for target matching. The reason of the failure can be due to the sudden depth changes on the scene, and also, depth likelihood and joint color representation on the background region can have more similarity to the reference color representation. Otherwise, in general, depth integration can help to improve the robustness of SOAMST under changing illumination conditions.

**Figure 11:**
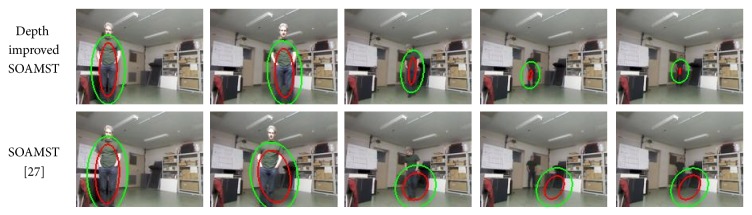
Tracking results for SOAMST and depth likelihood map supported SOAMST.

In Figure 11, images of the tracking results of SOAMST and depth likelihood map based improved SOAMST models are given. It is obvious that depth data integration to weight updates improves the tracking results significantly while preventing the failure cases as in the last two columns of Figure 11. But, still, if the color distribution model is not convenient and the depth values of the subject and background are similar, there can be still false detection or positions, scale, and orientation errors (see Figure 11 4th column of the depth improved tracking result) on the selected region.

In sum, limitations and optimal condition of the SOMAST [27] color tracker should be examined under different illumination and distance conditions to fully take advantage of depth data integration. Therefore, the experiments with various lux values and distance conditions, regarding the light strength, were tested on SOAMST [27] for further analysis by examining the color features and visual tracking.

### 3.2. Illumination Experiments for Visual Tracking

In this part of the work, we made several experiments by modifying the light conditions and distance between the robot and the subject. The aim of these experiments is to observe the impact on the visual tracking due to the changes in the color information produced by the different light conditions. Depending on the changes of the color, the robot may fail with possible false tracking. Using a light meter, illumination values at different distances were tested using the SOAMST [27] visual tracking algorithm. So, it is possible to see which lux (quantitative measurement of the light conditions) values provide good or bad tracking results with the given system.

#### 3.2.1. Experiment Setup

**Figure 12:**
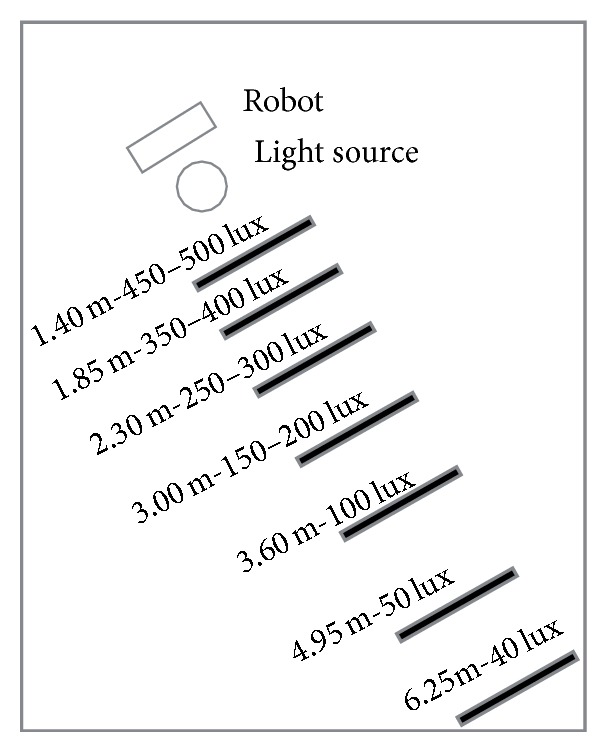
Illumination experiment setup.

The experiments for the illumination tests have been done in the scenario presented in Figure 12. The robot, with the Kinect camera, is in charge of recording the video and it is located in one of the corners of the laboratory close to the light source. A halogen lamp is the only light source used in the room.

**Figure 13:**
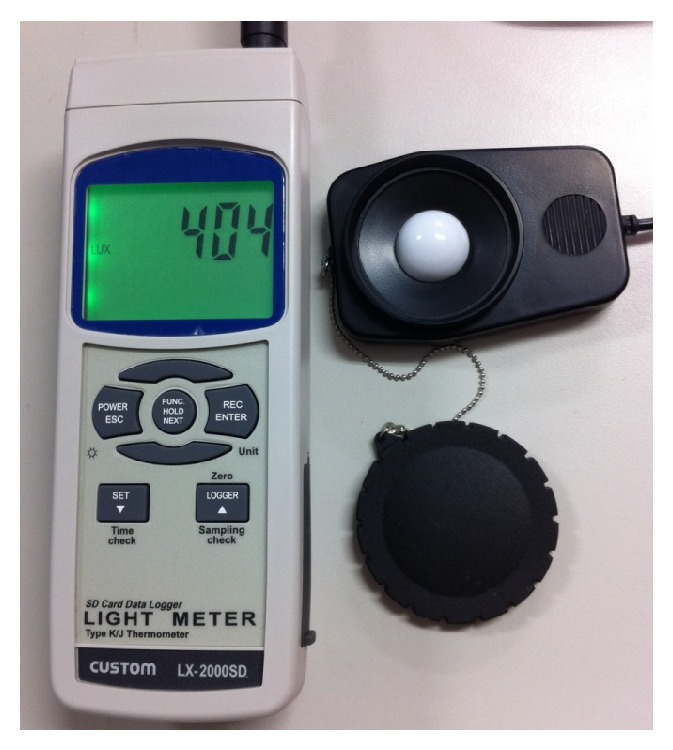
Illumination sensor: CUSTOM LX-2000SD light meter.

Different spots to evaluate the tracking system have been established based on the luminosity ranges measured by the illumination sensor, CUSTOM LX-2000SD light meter (Figure 13). For example, the first spot has an illumination value between 450 and 500 lux and it is located at 1.40 meters from the camera (Figure 12), and the values at 4.95 and 6.25 meters are 50 ± 15 lux and 40 ± 5 lux, respectively (Figure 12).

#### 3.2.2. Color Histogram Analysis with Changing Conditions

For this experiment, the robot with the camera is fixed at the position presented in Figure 12. It records a video of the immobile subject for each of the marks to see the variations in color representation by checking the histogram representation.

**Figure 14:**
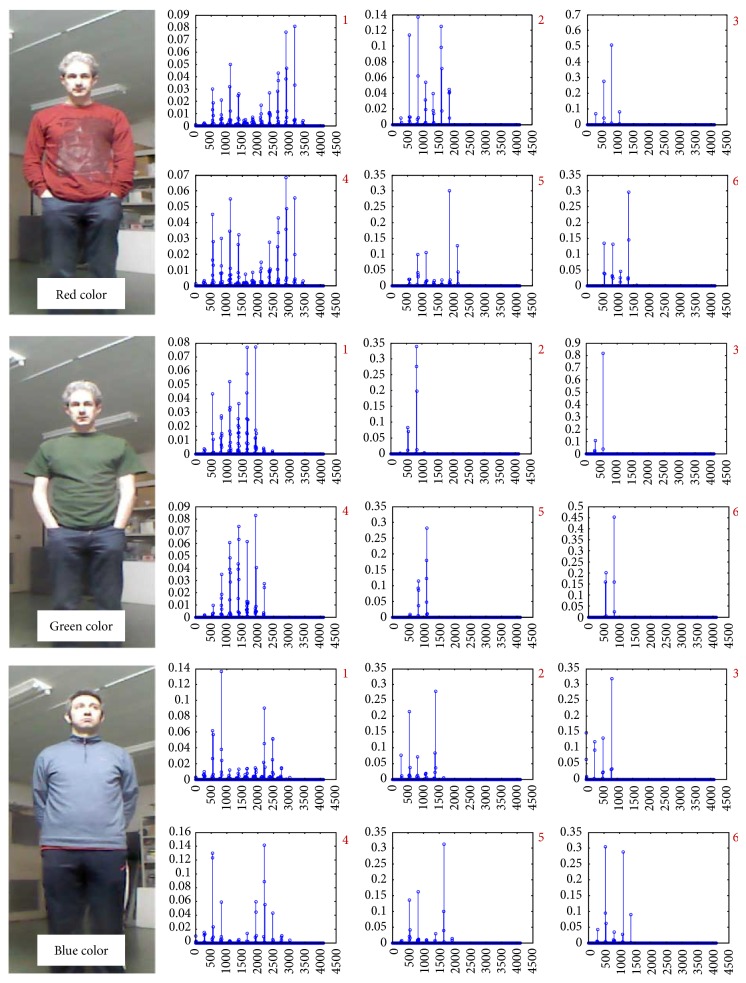
Target feature analysis for different colors with normalized histogram

The color histogram results are presented in Figure 14. Histograms 1, 2, and 3 show the information when the subject is at the marks at the distance of 1.85, 3.60, and 6.25 meters, and in Figure 14, graphs 4, 5, and 6 demonstrate the histograms at the same marks while the robot has been moved from the initial position closer to the subject, within the distance of around 1.5 to 2 meters.

As it can be seen from the preliminary analysis, light condition and the distance between the subject and robot have a great impact on the reference target model based on normalized color histogram for visual color tracking algorithm due to the fact that these conditional deviations may change the texture details of the region selected on the subject. However, for a better understanding of the limitations, in this work, additional experiments were done to investigate these conditions with SOAMST [27] color based region tracker.

Hence, at this scenario, two more different tests have been done. (i) One test was to study visual tracker while the robot and subject move. (ii) Then, visual tracking performance was tested when the robot is in a fixed position and just only the subject moves. Both experiments were done with two different scenarios where two different region templates were tried in each case to see the performance difference by using one- and two-color region models. The following sections present the experiments and their results of these tests.

#### 3.2.3. Robot and Subject Moving

At this experiment, both the robot and subject are moving. The robot moves while trying to keep a constant distance within an interval from 1.5 to 2 meters. The subject moves in the marks set up at the distance of 1.85, 3.60, and 6.25 meters where there are three different light conditions: around 350–400 lux, around 100 lux, and below 50 lux, respectively.

These experiments make it possible to determine which lux values are in the safe or critical zone for region tracking models. The experiments on the tracking were done using three different color representations separately: (a) red, (b) green, and (c) blue, while testing just one color for subject representation.

**Figure 15:**
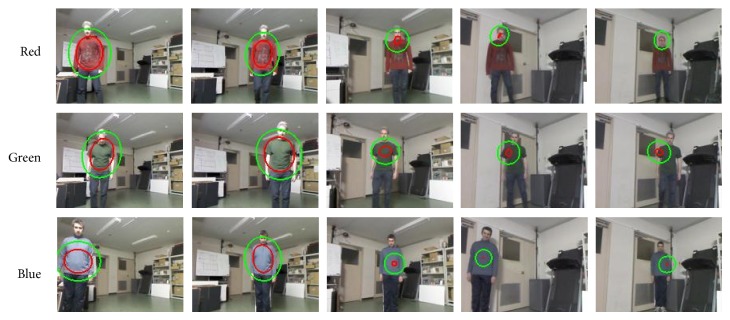
Moving robot condition: tracking results with one color representation.

Figure 15 and Table 3 present the tracking results for each color space. In Figure 15, the green ellipsoid region is the search area to match the target model, and the red ellipsoid region is the detected target region where centroid of the target region defines the tracking point of the current frame. So, tracking success or failure is decided whether the center of the target region is on the subject or not.

Since, during the subject's motion, the distance between the robot and the subject has been the same in all the experiments, one should expect that search and tracked regions by the SOAMST [27] would be similar too; however, there are high deviations for the matched region because the illumination value changes. The SOAMST [27] algorithm calculates the covariance based on the current search region and reference target distribution in which this matrix based on the second order moments can represent the height, width, and orientation variance of the ellipsoid that defines the tracked region on the subject [27]. So, depending on the orientation of the tracked ellipsoid region, these high deviations on the region representations can be visible either on the width or on the height scale as in Table 3. In particular, the lux values around 350–400 (frames around 1 to 400) keep the subject tracking region similar; however, when the subject moves to darker region, around 100 lux (around frame 600 to 1200 in Table 3), target model and current matching are not consistent enough due to the loss of information in the normalized color histogram, so tracking quality decreases as this problem was also presented in Figure 14 priorly.

But, despite the loss of information, tracking can be still performed. However, if the light on the subject goes under 50 lux, it is appreciated that the selected target and tracked region are not consistent anymore (last two columns of Figure 15). Moreover, for the blue color, the tracking algorithm fails to calculate scale and orientation changes with less than 50 lux. For red and green colors, the tracking is possible but the tracked regions consistency decreased significantly.

**Figure 16:**
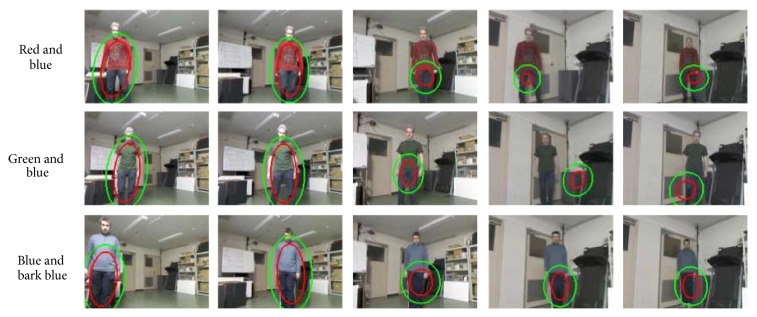
Moving robot condition: tracking results with two-color representation.

In addition to the one-color based tests, the other test has been done using two colors: red and Blue, green and blue, and blue and dark Blue. The results of this experiment are given in Figure 16 and Table 4. The use of two colors increases the initial target region on the subject (see the first columns of Table 4) when compared to the one-color model situation. The impact of the light conditions has been proven to be similar to the previous scenarios when only one-color representation is used for target model.

#### 3.2.4. Robot at a Fixed Position and Subject Moving

For this experiment, the robot with the camera is in a fixed position, the same position as demonstrated in Figure 12. The camera records data while the subject is moving at the marks located in a distance of 1.85, 3.60, and 6.25 meters. It should be noted that, while the subject is moving, the same distance will not be preserved between the subject and the robot as in the previous experiment. The purpose of this experiment is to evaluate the impact on the tracking system when modifying distance and illumination together by observing the relation between these two parameters.

When the distance was kept in a constant interval between the subject and the robot, tracking could be done even at low lux light conditions such as 100 lux even though it was not the desired case. While using just one-color representation, tracking performance was acceptable if the light conditions were above 100 and distance was between 1 to 3 meters.

**Figure 17:**
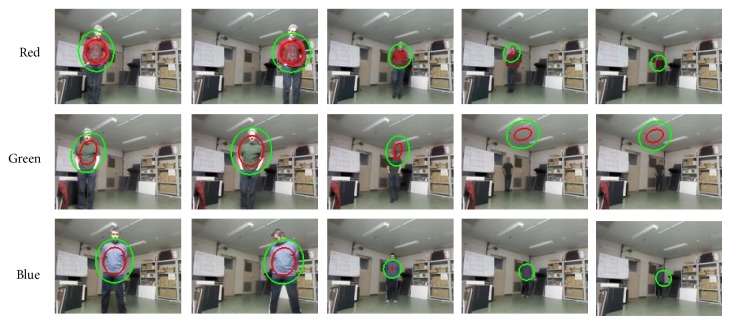
Fixed robot condition: tracking results with one-color representation.

**Figure 18:**
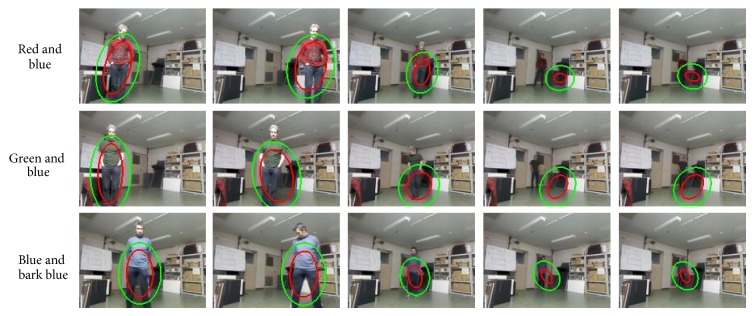
Fixed robot condition: tracking results with two-color representation.

On the other hand, when the robot is not moving and the subject is 3.6 meters distance, which has around 100 lux value, green color based tracking (see Figure 17 4th column) failed because target color model matched with an irrelevant region, which also had similar color to the reference target model, inside the search area. Finally, red and blue cases barely could continue tracking the subject (Figure 17). So, the light conditions below 50 lux with a distance higher than 5 meter are not sufficient to rely on color based tracking for all tested color samples. In addition, for the two-color and the fixed robot experiments, subject tracking fails for all the cases when the light condition is less than 100 lux and the distance between subject and robot is greater than 3.6 meters (Figure 18).

## 4. Robot Behaviour Experiments with Different Robot Motion Parameters While Tracking a Subject

After the visual tracking experimental analysis based on illumination and distance factors, the results state that some constraints should be applied to the robot tracking such as stable light conditions and reliable distance between subject and robot. Then, with these constraints, robot tracking can be tested for further improvements.

With the goal of making the system more robust, the objective of these tests is to analyze two main parameters that can significantly affect the motion of the robot for tracking a subject while it is moving and changing his trajectory and to establish the optimal values for them. The two parameters are the speed of the robot and the inflation radius of the obstacle definition that can affect the path planning during tracking task.

In addition, regarding the at-home biomonitoring application, the aim is to follow and recognize the behavior of people with walking difficulties such as the elderly and motor-function impaired people. Since the robot can track the elderly or impaired walking patterns easier compared to normal walking and the speed of the targeted subjects is slower than 3 km/hr, which is the lower limit of self-paced normal walking, the experiments regarding robot velocity and inflation ratio were done with normal walking pattern only.

The speed parameter is set up as the maximum speed of the robot. This parameter has effects on keeping the target in the visual scene and on the required time to slow down or stop. The inflation radius affects the path of the robot while tracking the subject due to the fact that it expresses the obstacle definitions or available free space for the robot.

This section describes the experiment setup and the two tests that have been performed: (i) the collision speed and (ii) the robot performance tests. The first test aims to establish the maximum speed for the robot that prevents it to collide with the user considering the subject and robot safety criteria. The second one analyses the speed and the obstacle inflation radius of the robot to evaluate how they change the performance of the tracking process.

### 4.1. Experiment Setup

**Figure 19:**
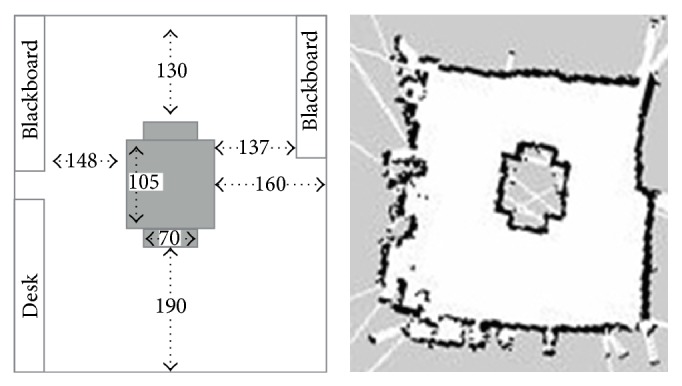
Sample for experimental layout (the numerical values in centimeters) and global map.

**Figure 20:**
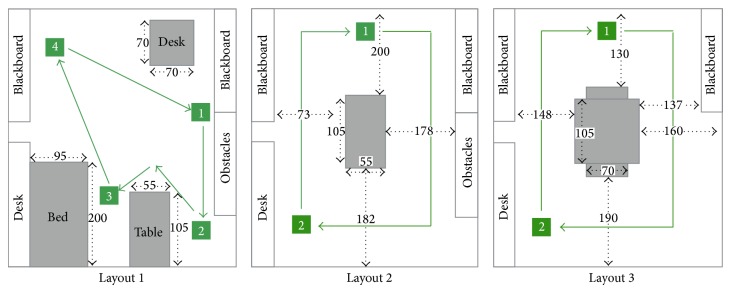
Experiment layouts and subject tracks (the numerical values in centimeters).

The experiment setup is done by defining a set of configuration parameters and building the laboratory map. To set up the experiment, the first step is to create the laboratory maps using mapping function of the robot as in Figure 19 for the three different layouts used in these experiments. Also, in our experiment, the same room was used in all tests but different layouts also were tested by making smaller or larger free space for checking robot behaviour in different situations. In these layouts, setting the experiment in the same room or in a different room was not very crucial since the important point is testing the robot motion in different situations with various free space options. These layouts (see Figure 20) may not present a problem for the system for autonomous navigation, but they do so for performing navigation with subject tracking task based on the visual tracking feedback. For the visual tracking system, the robot must keep a direct visible line with the subject for vision feedback, and also, it has to update the path to follow the subject based on the position of the subject on global map.

The layouts have been designed to represent different situations that may occur in the real environments. The first layout represents a room with no obstacles in the center. All the obstacles are in the borders of the room. In the second and third scenarios, there is an obstacle in the center of the room that makes it more difficult for the robot to move as it is forced to follow the subject where there is only one direct path available. The main differences between second and the third layouts are the size and shape of the central obstacle. In the third layout, the obstacle is bigger reducing the available path for the robot.

Once the physical layout is already deployed in the laboratory, the next step is to build the map that the robot uses in the tracking process. The map is built as described in [23–25]. In Figure 19, the image at the right is an example to present the robot map built from the laboratory layout. The areas in black represent the obstacles for the robot and the white areas are safe for the robot to move. As it can be seen, the robot is free to move in the room except in the area delimitated by the obstacle in the middle of the room.

Two parameters, for robot decision on moving to follow the subject, are common to all the experiments presented in this paper. The maximum distance between the robot and the subject is set to 1.2 meters. This means that the robot starts moving when the distance to the subject is higher than this value; in another case, the robot is able to track the subject without moving.

The maximum distance has been established based on the results of the illumination tests. As stated in the previous experiments for a distance higher than 3.6 meters, color based visual tracking system does not provide reliable results due to illumination and distance variations that cause low similarity or texture details. A value of 1.2 meters provides some reaction time to the robot to start moving when the subject starts moving while keeping the distance between them lower than 3.6 meters. Besides, the robot considers that the subject is moving when it covers a distance change of at least 20 cm on the global map as described in the activity recognition experiments.

As stated previously, there are two important parameters for robot motion, included in this part of the paper, which have been studied in order to determine their impact for the tracking and movement of the robot. These parameters are the speed of the robot and the inflation radius of the obstacles. At the system (ROS) for the robot motion (Figure 2), these parameters are modeled with the following variables.

- Maximum speed of the robot: the speed of the robot is controlled by the max_vel_x parameter. The values that this parameter can take represent the maximum forward velocity, in meters/seconds, of the robot. This parameter is included in the configuration file costmap_common_params. Yaml.
- Inflation radius: this parameter represents the radius, in meters, to which the map inflates obstacles cost value. This parameter is included in the configuration file costmap_common_ params.yaml.

### 4.2. Collision Test Results

Once the experiment has been set up, it is possible to start the tests to analyze the impact of the speed and the obstacle inflation radius. While increasing the speed of the robot, a new problem appears because of the robot deceleration. At higher speeds, robot needs more time to stop. If the speed is too high, the robot may not have enough distance with the subject, and they may collide.

The objective of the collision speed test is to determine the maximum speed of the robot that implies no collision with the subject. To perform these tests, the subject walks from the starting point to the mark “2” of the map (Figure 20 layout 3). The speed of the subject has to be, at least, faster than the robot. When the subject reaches the mark “2”, subject waits until the robot stops.

Table 5 presents the results of the test by modifying the speed of the robot and the obstacle inflation radius. The speed is decreased from 0.70 meters/sec until the robot does not crash with the subject. In our tests, the obstacle inflation radius seemed to have no influence on the results of the collision tests such that using a low inflation radius value, 0.05, or a higher one, 0.35, does not affect the collision test results.

### 4.3. Robot Performance Test Setups

Speed and obstacle inflation radii are two parameters that affect the trajectory of the robot during subject tracking task. This section describes the tests to study the impact of both parameters on the performance of the tracking process.

The maps used for these experiments are the ones presented in Figure 20. With the layouts of these maps, two different tests are defined.

- Nonstopping test: this test only includes the third layout in Figure 20. At this test, the subject walks faster than the normal speed, around 1 step per second. He moves from the mark “1” to mark “2” and then to the mark “1” to start over.
- Stopping tests: these tests have been done on each of the three layouts shown in Figure 20. The subject moves from one mark to another by following the paths presented in Figure 20 in the order of the destination numbers, and, at each destination point, the subject stops and waits for 3 seconds.

Once the subject has completed the number of turns established for each experiment, the test is over and the results are logged. The results of the experiments contain whether the robot was able to complete the track, the time to complete the path, and the path followed by the robot.

There are three possible reasons about why the robot was not able to complete the track:

- the robot lost the tracking of the subject;
- the robot crashed with any of the obstacles of the room;
- the robot got stuck for more than 30 seconds.

All tests have been done by modifying the different values for the maximum robot speed and inflation radius parameters. The rest of parameters in the localization process remain the same for all the tests.

### 4.4. Nonstopping Tests

The nonstopping tests represent an unnatural situation as the subject is continuously moving around one obstacle at fast speed. This scenario can barely happen in the real life. However, the objective of this scenario is to analyze the behaviour of the tracking system when the robot is pushed to move and turn fast. It also represents a challenge for the Kinect moving camera, as the synchronization between the quick movement of the robot and the moving platform is not an easy task. Due to this fact, it is possible to loose the track of the subject.

Faster speeds also mean more vibration for the robot, which can contribute to loosing the localization of itself on the map. When the localization is lost, the robot can collide with the obstacles in the room.

As it was previously proven in Section 4.2, the maximum speed to prevent the robot to collide with the subject is 0.60 m/s. But for these tests, it is possible to increase the speed of the robot over that speed as the subject is walking faster so there is no risk that the robot could collide with him. So the speed values tested in this scenario are 0.50, 1.00, and 1.50 m/s, respectively, and the obstacle inflation radius goes from 0.05 to 0.35 meters in increments of 0.1 meters.

For this experiment, the subject turns four times around the obstacle, starting from “subject start” point and finishing at mark “1.” To complete one round to the circuit, at the established subject speed, it takes around 15 seconds. This means that the each test for a given speed and inflation radius should take around 1 minute for the whole 4 rounds.

The results of the experiment are shown in Table 6. The values in the fields of the table indicate if the robot was able to complete four rounds in the circuit and, if applicable, the extra time over the normal time to complete the experiment. When the total time is much higher than 60 seconds, it means that the robot got stuck, but it was able to get back to the track and the experiment can be continued.

### 4.5. Stopping Tests

These tests were applied to the three different layouts and tracks as presented in Figure 20. All the tests were made for 5 turns around the room where the subject stops in the different points that are marked on the tracks as shown in Figure 20.

For each layout, the maximum tested speed is 0.60 m/s, which is the maximum speed for the robot that prevents the collision with the subject as proven in the previous section. Other tested values are 0.50 and 0.40 m/s. In addition, the inflation radius has also been modified for these experiments, in which it takes values from 0.05 to 0.45 meters in increments of 0.1 meters.

The results of the experiments are shown in Tables 7, 8, and 9. The values in the fields of the table indicate whether the robot was able to complete 5 rounds to the track and also whether the robot got stuck, but it was able to recover within the 30 seconds previously specified.

For the experiments performed in the two first layouts (see the results in Tables 7 and 8), the robot did not have any problems to complete the tracks. For the second layout, it got stuck sometimes, but it was able to recover the tracking and ends the tests with success.

**Figure 21:**
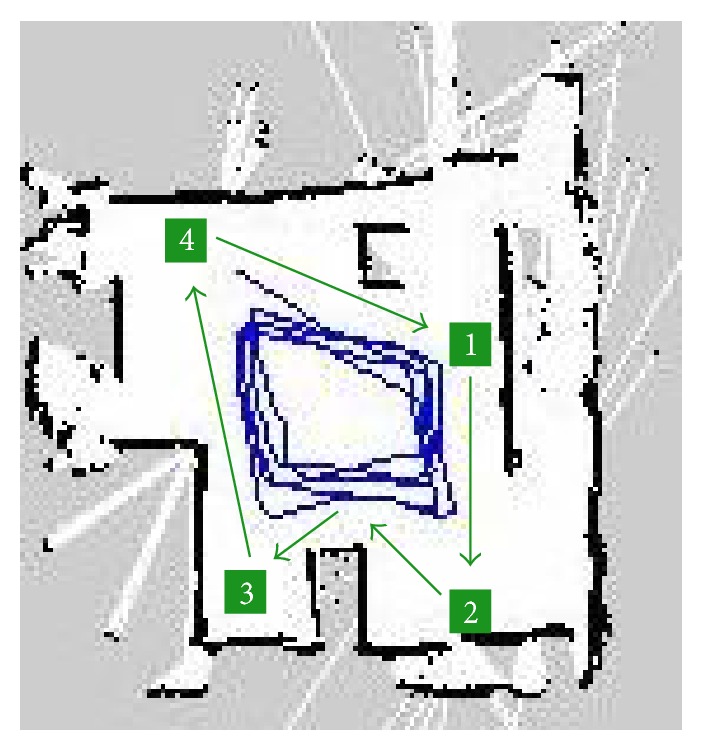
Track for layout 1 and robot path during the experiment.

For the first layout (Figure 20), as presented in Table 9, the results show that the robot has no problem to track the subject in any of the combinations of speeds and inflation radius. The main reason of the good results is that, in this kind of layouts, the robot can move in the center of the room. The robot is not forced to follow the same path as the subject, but it can track him/her just by moving in the center of the room avoiding most of the obstacles that can cause a collision. Blue lines in Figure 21 express the path followed by the robot while tracking the moving subject with the path marked with green lines.

The second layout (Figure 20) presents a scenario where an obstacle is in the middle of the room. This obstacle represents furniture such as a table and a desk. With this layout, we are preventing the robot from have a comfortable position in the middle of the room as in the first layout, and by this way, we force it to move around the room following the subject. In this layout, the empty space around the obstacle is high, and it allows the robot to move around without serious difficulties. For some of tests in this condition, the robot was stuck, but it could recover within the given 30 seconds.

The third layout (Figure 20) represents the most restrictive scenario where the robot is forced to follow a narrow path while tracking the subject. The shapes of the corners of the obstacle present also an extra difficulty for the robot. The lowest speed that has been tested was 0.4 m/s. For this speed value, there was no success in any of the tests independent of the inflation radius value. The reason of why the robot does not complete the track is due to the fact that robot was stuck during tracking, especially because of entering the corner regions that restricted the robot to define a free path to move. In this layout, faster speeds increased the success ratio of the experiment. For the 12 tests made, 6 of them present good results while the robot was not able to complete the 10 rounds of the track in 8 of the remaining results. As presented in Table 9, the results are not as good as for previous experiments. The narrow paths and obstacles drastically reduce the success rate of the experiments.

## 5. Experiments on the Activity Recognition Algorithm

After completing visual and robot tracking experiments, finally, this section expresses the experiments on activity recognition, especially walking activity detection for data recording to support possible health care assistance to the subjects. For the experiments, first, data were recorded from two subjects with their normal walking and two types of simulated walking patterns such as impaired and elderly walking, where elderly and impaired walking conditions were simulated by wearing tools as demonstrated in Figure 10.

For each case, 1000 frames were used to test the activity recognition algorithm as given in Table 1 of visual tracking data, in which subjects walk or stand inside the room continuously. In our work, walking activity detection has higher priority on other activities since the walking patterns can be analyzed whenever necessary, especially for the elderly and impaired people at-home health care support. It is important to record the data during the subjects' walking activity, and it can be achieved for different subjects and walking patterns robustly. Hence, in this part, we tested the recognition rate of the algorithm for different walking patterns separate from other activities even though including the static activities would increase the accuracy due to high detection rate of static actions.

In the experiments, person specific parameters for activity recognitions are decided for the subject 1 and impaired walking case initially, and these selected parameters are applied on all cases without any change. Impaired walking (IW) is the slowest motion among all cases so velocity parameter is defined according to IW where it is given as 20 cm per 10 frames that is around 0.2 m/s for the current system.

Table 10 gives the activity recognition results for the recorded data described in Table 1. The results show that walking condition can be recognized with high accuracy for various walking patterns even though the same heuristics were used for all cases. In both subjects, impaired walking tests have the best accuracy on detecting the difference between standing and walking activities around 98%. Normal walking of subjects had the lowest accuracies around 93% and 95%; however, these results are still in acceptable accuracy, and overall accuracy for all data is more than 96%, which is quite high considering the usage of same heuristics in each case.

Moreover, our analysis on false recognition generally includes the cases during transition states such as: (i) start of walking from standing, (ii) transition from walking to standing condition, and (iii) rotation of the subjects around themselves with high position changes. On the other hand, the accuracy of correctly detected walking activity is more than 99% and is almost 100% when the subjects are in the state of continuous walking motion without transition condition from one state to another. Therefore, it can be concluded that the proposed activity recognition algorithm is a reliable approach to record the walking activity cases for the purpose of further analysis as an assistive technology for at-home biomonitoring systems.

In addition, we also made experiments on other activities including, standing, sitting, bending, lying down, on cycling, and falling, where falling is another important case such as walking in at-home monitoring applications. In all these experiments, since the activities such as standing and sitting were static cases without any motion except falling activity, we started recognition when the activity was in its state without transition periods as in changing from one activity to another. For example, the frames during transition from standing to sitting, standing to bending, and so forth were not count because these transitions were not the actual activity of standing, sitting, bending, or lying down cases. Also, for the falling case, the activity was classified as falling continuously until the subject moved and changed the activity state to other activities.

In Table 11, the recognition results for various activities were given, where 1000 frames were tested for each activity. As it can be seen that static behaviors such as standing, sitting, and lying down yielded 100.00% accuracy without any false recognition since these activities were very stable due to their static nature. Bending had 3 false recognition cases among the 1000 frames, and this is probably because of the large amount of motion and the change in pose and height during each bending activity, which might have caused misclassification to similar or transition activities, such as sitting or standing, and their transition states. Again, with the assistance of localization information, activity on cycling machine could be recognized at 100.00% accuracy. Also, falling activity was recognized with more than 99% accuracy, which is crucial to detect this activity on emergency actions for the people living alone. And, over all recognition rate was obtained as 99.80% from 6000 frames for the tested activities shown in Table 11.

**Figure 22:**
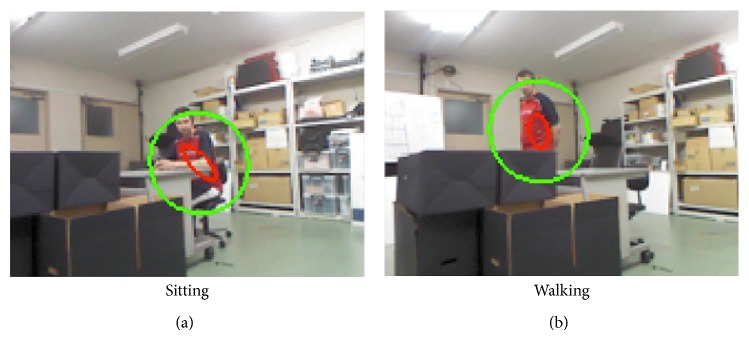
Sample robot system tracking and activity recognition results with partial occlusion cases for (a) sitting and (b) walking activities.

In addition to these results, in Figure 22, two cases with occlusion of the lower limbs were given, and these two activities were correctly classified by the proposed activity recognition model. Many algorithms, including our previous implementations, [5, 8] handle the activity recognition by taking advantage of features extracted from lower limbs and upper body. However, when there is an occlusion on lower limbs, activities such as walking can not be recognized accurately, not being able to extract any observable features from lower limbs [5, 8]. Therefore, our proposed model makes full use of the tracking position, height of the tracked subject, and global location of the tracked subject as explained in methodology section for activity recognition (Section 2.5). With the information, even though there is not clear information about lower limb joints (Figure 22(a)) or the partial occlusion on both upper body and lower body (Figure 22(b)), walking activity could be detected accurately only if we know that the height of a subject is comparable to the height of walking pose and the subject's global position is changing on global map. And, our walking tests in Table 10 demonstrate that walking can be recognized with high accuracy using person specific features and localization assistance. In sum, it can be seen that the proposed system can handle occlusion situations when the subject is accurately tracked as in the examples shown in Figure 22.

## 6. Discussion

This section provides an analysis of the results presented in the previous experiments, the illumination and the speed and obstacle inflation radius results, in order to determine the best setup for the system.

### 6.1. Visual Tracking and Illumination Results

First of all, the experiments for the visual tracking demonstrate that integration of the depth information to handle the illumination variations improved the tracking results obviously. It means that multiple sensory information fusion is required for a robust indoor tracking to achieve at-home monitoring mobile robot. Hence, to be able to handle various illuminations, different sensor types can support the system such as the infrared sensors in the case of low illumination or dark room conditions.

Based on the results of the performed tests, it is possible to state that color information may not be reliable all the time during real-time tasks considering the subject that is tracked by the mobile robot during daily activities. So, it is beneficial to find out limitations of the color tracker used in our system and safe regions to utilize the visual tracker relying on color considering the distance and illumination conditions.

In sum, this experimental analysis concludes that some certain conditions should be provided at the environment for a reliable color region based visual tracking algorithm: (1) the illumination condition on the subject to be tracked should be greater than 100 or 200 lux for better results and (2) for the given lux and distance specifications, as in Figure 12, if the distance between robot and subject is able to keep the interval less than 3.6 meters, there is high chance of successful and continuous robot tracking supported by the visual tracking algorithm.

### 6.2. Speed and Obstacle Inflation Radius Results

The speed of the robot and the inflation radius are two important parameters to be considered when performing the tracking of the subject and the results of the activity may differ depending on the layout of the room. There are no studies that analyze parameters that may impact the behaviour of a robot when performing a vision tracking activity. Therefore, the objective of the experiments related to those parameters was to identify the speed range and the obstacle inflation radius where the tracking process is more stable with the given constraints of visual tracking algorithm.

The speed parameter is needed to be high enough to be able not to lose the subject but slow enough not to make the robot collide with the obstacles and/or the subject. When the speed of the robot is high, the robot covers more distance while stopping. This distance, while the robot is decelerating, can be not enough and may cause the collision with the subject or even with an obstacle. Using the results presented in Table 5, it is possible to determine the maximum robot speed to prevent the collision with the subject. The collision is inevitable when the max_vel_x parameter takes values over 0.65. Based on this results, the robot speed must be set at 0.60 m/s or even lower than that to prevent this from happening. The obstacle inflation radius has no influence on the experiments.

Experiments described in Sections 4.4 and 4.5 study the performance of the tracking system based on the speed and the obstacle inflation radius. With the results of these tests, it is possible to compare the path followed by the robot and to check if it is able to complete the specific track of the experiments.

As it has been mentioned, nonstopping tests do not represent the normal behaviour of the activity performed by humans at home. Table 6 shows the results of these nonstopping tests. As it is presented, the rate of success is quite low, especially when the speed is set to 1.00 and 1.50 m/s.

**Figure 23:**
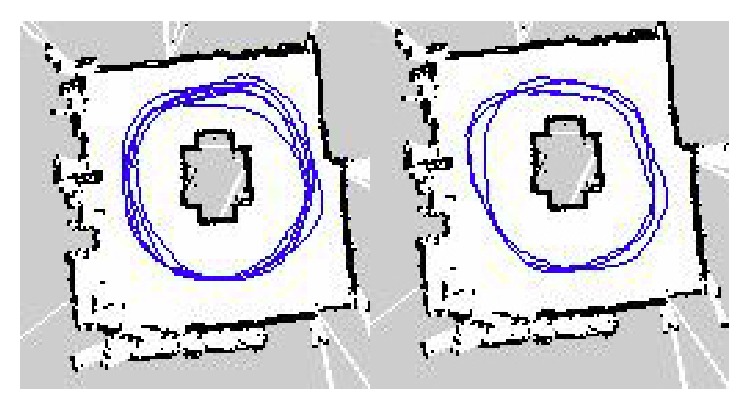
Track for 1.5 m/s speed and 0.05/0.35 of inflation radius.

It is possible to observe how the inflation radius affects the path followed by the robot. The influence of the inflation radius is presented in Figure 23. For both maps, the speed of the robot is set to 1.5 m/s. The map on the left presents the track of the robot for an obstacle inflation radius value of 0.05 and the one on the right for a value of 0.35. As it can be appreciated the track is softer when the inflation radius is lower. At the map of the right the curves when the robot is turning are sharp bends. A softer path makes the robot less suitable for loosing the track or the localization in the map. This is the reason why the results at Table 6 present good results for an inflation radius value of 0.05 but the results get worse when this value is increased.

Another interesting result presented in Table 6 is the time to complete the track when the speed is set to 0.50 m/s. The time values are higher for this value even when the subject is always walking at the same speed. Because the speed of the robot is not high enough, the robot sometimes got stuck for a few seconds. The subject has to wait for the robot to be released and that results in increasing the required time to complete the track.

The situation when the subject stops is different from the one previously exposed. People at home are used to walk and stop when walking in a room. The walk to a desk and stop for a moment to pick something and then start walking again and so on. This is the situation represented by these experiments, and this situation is more favorable for the tracking process. As the user stops for a short time, the robot can use that break to recover the lost distance and get a better position in the room. This allows for the use of lower speeds by increasing the reaction time of the robot.

In this part, we have analyzed three different scenarios representing three layouts with different characteristics. In an open layout, Figure 20—layout 1, when there are no obstacles in the middle of the room there is no restriction for the speed of the robot; the only limit is the maximum speed of 0.60 m/s that prevents the robot from crashing with the subject. The robot is able to perform its activity without any problem. Moving in the room center freely, the speed or inflation radius do not have any effect on the performance. The same results are obtained when the robot has a wide space to move around an obstacle (Figure 20—layout 2). The robot can follow the subject, and the success in all the tests states that any value in the range of the one tested provides good results.

When there is not enough space to move and/or when the shape of the obstacles is complex the speed and inflation radius for the robot motion have to be considered to guarantee the success of the tracking process. As a result of the experiments, only speeds over 0.40 m/s lead to success. Values around 0.50 m/s and inflation radius over 0.15 increase the success rate of the tracking activity even if it does not provide 100% success.

The exception for this speed range was when the inflation radius was set to 0.05 meters. Using this low value, the robot was not able to complete the track. Probably, with 0.05 meters inflation ration, the robot can get close to the obstacles and so this may make it easier for the robot to get stuck during subject tracking task. Indeed, the reason why the robot could not complete the track was always that the robot got stuck during navigation.

The success rate for these two speed parameters, when the inflation radius is over 0.05, is 5 tests out of 8. At these speeds the robot looks quite stable and the only reasons not to complete the track are that if it was stuck in some points of the track. But generally the parameter combination with a speed of 0.50–60 m/s and an inflation radius 0.15–0.45 offer the best results in complex scenarios.

As a summary, for open and wide areas, speed and inflation radius do not affect the performance of the tracking activity. But, for the not ideal conditions, if the room contains a narrow path of complex obstacles, the value of these parameters should be restricted to the range proposed in this paper. The results of the test show that setting the speed of the robot at 0.5 or 0.6 m/s and the inflation radius between 0.15 and 0.45 generates better results.

### 6.3. Activity Recognition Results

With mobile robot, if a robust visual tracking and robot based subject tracking is provided, the experimental results depict that it is possible to achieve activity recognition for different subjects and walking patterns using the same simple heuristics, which is crucial to record reliable data of the subjects.

Our previous works [5, 8] had activity recognition performances over 98% with high accuracy; however, these models required training data and tuning for each specific subject, which is difficult to handle each time when the subject is changed. Also, these calculations including the state-of-the-art models, and that generally requires whole body contour features for walking or dynamic activity detection. But, with the proposed model, the activities can be recognized under limited conditions such as partial occlusion.

Using the proposed model with simple person specific heuristics and localization assistance, we were able to obtain 98% activity recognition accuracy for all activities tested by 12000 frames even without using large amount of training data. The proposed algorithm does not require color image based feature extraction, which is also important by providing robust activity recognition under changing environmental conditions. Moreover, dynamic activities on static positions such as cycling on cycling machine can be detected also with the localization assistance provided by the subject global position.

It can be concluded that the proposed activity recognition algorithm is a reliable approach to record the walking activity cases for the purpose of further analysis as an assistive technology to at-home biomonitoring systems.

## 7. Conclusion

This paper has presented three main contributions: the proposal of a new activity recognition algorithm, the effect of illumination conditions in the visual tracking process, and the impact of parameters related to robot motion and path panning in the robot navigation behaviour when tracking a subject.

Based on the illumination tests it has been proven that the ambient illumination also affects the behaviour of the robot. The better results are obtained with illuminations of 100–200 lux or more. When this light condition is presented the robot can perform the tracking process with high chances of success. In addition to the light condition, the distance, which also effects the texture details obtained from the sensor, is an important factor, and better results are observed in the conditions that the subject to robot distance is less than 3.6 meters. Since the reliability of color based visual tracking is decreasing due to distance and illumination factors, a better approach for visual tracking task to work with changing illumination conditions should be modeled, for example, by switching from color to infrared sensor based tracking. In addition to visual tracking, robot existence as a assistive technology and its behaviour indoor environment should be studied qualitatively concerning the human feelings and psychological response to use the mobile robot systems.

Analyzing robot parameters for the scenarios used for the experiments presented in this paper, it has been shown that the two analyzed robot parameters, speed and inflation radius, can affect the behaviour of the robot while the robot is actively tracking the subject with visual tracking model. The experiments presented in this paper have to determine the best speed and obstacle inflation radius parameters for the tracking process. For simple scenarios, the impact of these two parameters is not relevant but for more complex scenarios the robot fails while tracking the subject for some parameters combinations. For these types of scenarios, the tests speeds of 0.5 and 0.6 m/s offered better results. Increasing the speed over these values provokes the collision of the robot with the subject. Decreasing the speed can make it easier for the robot to get stuck. At the recommended speeds, the obstacle inflation radius must be greater than or equal to 0.15 to offer better results.

The new localization assisted activity recognition algorithm has been integrated with the robot platform. This algorithm is faster and more robust, and it is effective in those cases where the lower limb body is not visible. It also does not required training data to operate. The results are promising for being able to detect walking activity for different subjects and walking patterns.

In summary, the contribution of this paper can be stated as (i) integration of a new localization assisted activity recognition algorithm in a mobile robot that tracks a subject; (ii) analyzing how the illumination conditions and robot motion parameters affect the tracking; and (iii) finding the optimal conditions for robot based subject tracking where the conditions can be listed as (a) quantitative light conditions (lux value), (b) robot to subject distances, (c) robot speed, and (d) map inflation radius during path planning and robot motion.

## Figures and Tables

**Algorithm 1:**
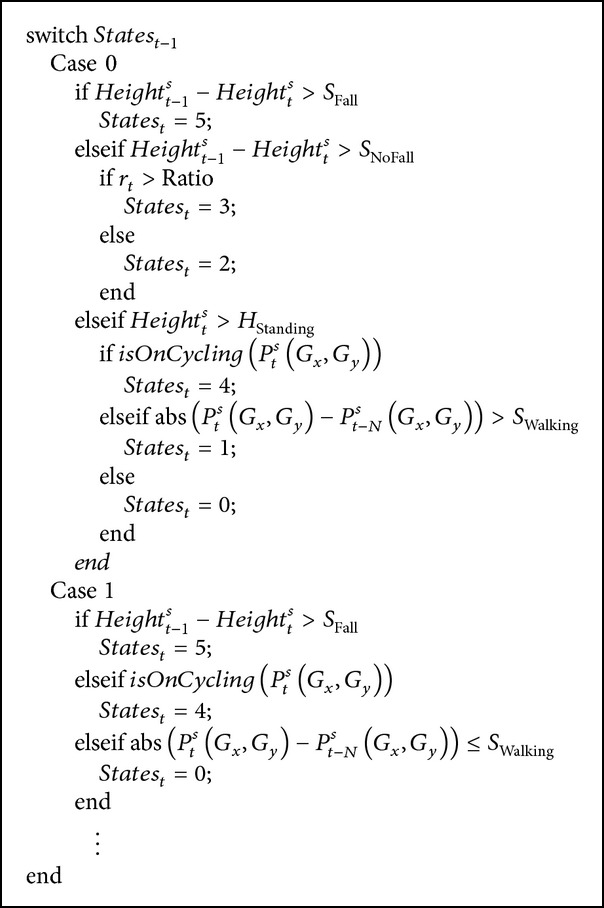


**Table 1:** Experimental data content.

Subjects	Walking case	Abbreviation	Number of frames
Subject 1	Impaired walking	IW	1000
	Elderly walking	EW	1000
	Normal walking	NW	1000

Subject 2	Impaired walking	IW	1000
	Elderly walking	EW	1000
	Normal walking	NW	1000

Total number of frames			**6000**

**Table 2:** Visual tracking results.

	Type	SOAMST [27]		Depth improved SOAMST	
		True	False	True	False
	IW	774	226	1000	0
Subject 1	EW	851	149	1000	0
	NW	899	101	1000	0

	IW	1000	0	1000	0
Subject 2	EW	1000	0	1000	0
	NW	998	2	914	86

Overall results		**5522**	**478**	**5914**	**86**

Accuracy%		92.03	7.97	98.57	1.43

**Table 3:** Moving robot condition: height and width variance of the tracking region with one-color representation.

Covariance	Red	Green	Blue
Region height variance	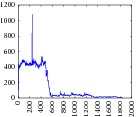	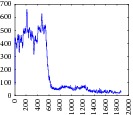	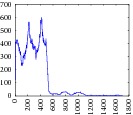

Region width variance	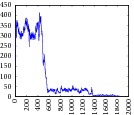	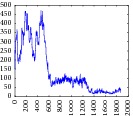	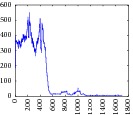

**Table 4:** Moving robot condition: height and width variance of the tracking region with two-color representation.

Covariance	Red & Blue	Green and blue	Blue and dark blue
Region height variance	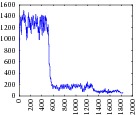	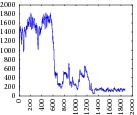	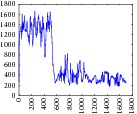

Region width variance	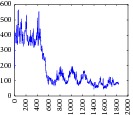	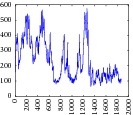	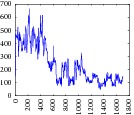

**Table 5:** Robot collision test results.

Distance/speed	0.05	0.35
0.70	Yes	Yes
0.65	Yes	Yes
0.60	No	No

**Table 6:** Robot tracking performance (nonstopping tests)^*^.

Speed/inflation radius	0.05	0.15	0.25	0.35
0.50	Yes (+30)	No^3^	No^3^	Yes (+40)
1.00	Yes	No^2^	No^2^	No^2^
1.50	Yes (+15)	No^2^	No^3^	No^2^

^*^Reasons not to complete the track: Stuck^1^, Crash^2^, and Lost^3^.

**Table 7:** Robot tracking layout 1 (stopping tests)^*^.

Speed/inflation radius	0.05	0.15	0.25	0.35	0.45
0.60	Yes	Yes	Yes	Yes	Yes
0.50	Yes	Yes	Yes	Yes^4^	Yes^4^
0.40	Yes	Yes	Yes^4^	Yes^4^	Yes^4^

^*^The index (4) indicates that the robot got stuck but recovered.

**Table 8:** Robot tracking layout 2 (stopping tests).

Speed/Inflation radius	0.05	0.15	0.25	0.35	0.45
0.60	Yes	Yes	Yes	Yes	Yes
0.50	Yes	Yes	Yes	Yes	Yes
0.40	Yes	Yes	Yes	Yes	Yes

**Table 9:** Robot tracking layout 3 (stopping tests)^*^.

Speed/inflation radius	0.05	0.15	0.25	0.35	0.45
0.60	No^1^	Yes^4^	Yes	No^1^	Yes
0.50	No^1^	No^1^	Yes^4^	Yes^4^	Yes
0.40	No^1^	No^1^	No^1^	No^1^	No^1^

^*^The index (4) indicates that the robot got stuck but recovered. The index (1) for not complete track cases indicates that robot got stuck and failed to track.

**Table 10:** Activity recognition results for different types of walking patterns.

	Type	True	False	Accuracy%
	IW	984	16	98.40
Subject 1	EW	974	26	97.40
	NW	947	53	94.70

	IW	976	24	97.60
Subject 2	EW	963	37	96.30
	NW	931	69	93.10

Overall performance		**5775**	**225**	**96.25**

**Table 11:** Activity recognition results for different activities.

Activity	True	False	Accuracy%
Standing	1000	0	100.00
Sitting	1000	0	100.00
Bending	997	3	99.70
Lying down	1000	0	100.00
Cycling	1000	0	100.00
Falling	991	9	99.10

Overall	**5988**	**12**	**99.80**
